# Observations on the Cerebral Effects of Refractory Intracranial Hypertension After Severe Traumatic Brain Injury

**DOI:** 10.1007/s12028-019-00748-x

**Published:** 2019-06-25

**Authors:** Joseph Donnelly, Peter Smielewski, Hadie Adams, Frederick A. Zeiler, Danilo Cardim, Xiuyun Liu, Marta Fedriga, Peter Hutchinson, David K. Menon, Marek Czosnyka

**Affiliations:** 1 Brain Physics Laboratory, Division of Neurosurgery, Department of Clinical Neurosciences, Cambridge Biomedical Campus, University of Cambridge, Cambridge, UK; 2 Department of Anaesthesiology, Level 12 Auckland Support Building, Auckland City Hospital, University of Auckland, 2 Park Road, Grafton, Auckland, New Zealand; 3 Division of Neurosurgery, Department of Clinical Neurosciences, Cambridge Biomedical Campus, University of Cambridge, Cambridge, UK; 4 Division of Anaesthesia, Department of Medicine, Addenbrooke’s Hospital, University of Cambridge, Cambridge, UK; 5 Department of Surgery, Rady Faculty of Health Sciences, University of Manitoba, Winnipeg, Canada; 6 Biomedical Engineering, Faculty of Engineering, University of Manitoba, Winnipeg, Canada; 7 Department of Human Anatomy and Cell Sciences, Rady Faculty of Health Sciences, University of Manitoba, Winnipeg, Canada; 8 Department of Anesthesiology, Pharmacology and Therapeutics, Vancouver General Hospital, University of British Columbia, Vancouver, Canada; 9 NIHR Global Health Research Group on Neurotrauma, University of Cambridge, Cambridge, UK; 10 Institute of Electronic Systems, Warsaw University of Technology, Warsaw, Poland

**Keywords:** Traumatic brain injury, Intracranial pressure, Cerebral hemodynamics, Autoregulation, Cerebral oxygenation, Cerebral perfusion pressure, Intracranial hypertension

## Abstract

**Background:**

Raised intracranial pressure (ICP) is a prominent cause of morbidity and mortality after severe traumatic brain injury (TBI). However, in the clinical setting, little is known about the cerebral physiological response to severe and prolonged increases in ICP.

**Methods:**

Thirty-three severe TBI patients from a single center who developed severe refractory intracranial hypertension (ICP > 40 mm Hg for longer than 1 h) with ICP, arterial blood pressure, and brain tissue oxygenation (P_BT_O_2_) monitoring (subcohort, *n* = 9) were selected for retrospective review. Secondary parameters reflecting autoregulation (including pressure reactivity index—PRx, which was available in 24 cases), cerebrospinal compensatory reserve (RAP), and ICP pulse amplitude were calculated.

**Results:**

PRx deteriorated from 0.06 ± 0.26 a.u. at baseline levels of ICP to 0.57 ± 0.24 a.u. (*p* < 0.0001) at high levels of ICP (> 50 mm Hg). In 4 cases, PRx was impaired (> 0.25 a.u.) before ICP was raised above 25 mm Hg. Concurrently, P_BT_O_2_ decreased from 27.3 ± 7.32 mm Hg at baseline ICP to 12.68 ± 7.09 mm Hg at high levels of ICP (*p* < 0.001). The pulse amplitude of the ICP waveform increased with increasing ICP but showed an ‘upper breakpoint’—whereby further increases in ICP lead to decreases in pulse amplitude—in 6 out of the 33 patients.

**Discussion:**

Severe intracranial hypertension after TBI leads to decreased brain oxygenation, impaired pressure reactivity, and changes in the pulse amplitude of ICP. Impaired pressure reactivity may denote increased risk of developing refractory intracranial hypertension in some patients.

## Introduction

Raised intracranial pressure (ICP) can occur due to an expanding mass lesion or due to increases in volume of any of the vascular, cerebrosipinal fluid (CSF) or parenchymal compartments within brain [1]. The detrimental effects of raised ICP are twofold. One is the development of transtentorial pressure gradients that can cause focal ischemia of vital brain stem centers leading to rapid death. The second is that increased ICP causes an increase in cerebral venous pressure by compression at the level of the bridging veins, leading in turn to a decrease in cerebral perfusion pressure (CPP) and, if CPP decreases below the lower limit of autoregulation, global cerebral hypoperfusion [2, 3].

Surprisingly, the cerebral physiological sequelae of raised ICP secondary to traumatic brain injury (TBI) are unclear. As previously shown, increased ICP decreases CPP, which with dysfunctional autoregulation, causes a decrease in cerebral blood flow, raises arterial blood pressure (ABP), and modifies the ICP pulse waveform [4]. However, whether these findings translate to the complex situation of raised ICP after severe TBI is uncertain.

In this study, we sought to describe the cerebral oxygenation, cerebrovascular pressure reactivity, and ICP pulse amplitude response to severe and sustained intracranial hypertension after severe TBI.

## Methods

### Study Design and Setting

This study was conducted as a retrospective analysis of a prospectively maintained database cohort (1992–2017), in which high-frequency physiological monitoring data had been archived. Monitoring of brain modalities was conducted as a part of standard patient care and archived in an anonymized database of physiological monitoring. Data on age, injury severity, and clinical status were recorded at the time of monitoring on this database, and no attempt was made to re-access clinical records for additional information. Since all data were extracted from the hospital records and fully anonymized, no data on patient identifiers were available, and therefore, formal patient or proxy consent and institutional ethics approval were not required.

### Participants

TBI patients with a clinical need for ICP monitoring and computerized signal recordings were included for analysis. A total of 1146 head-injured patients admitted to the Addenbrooke’s Hospital Neurocritical Care Unit between 1992 and 2017 were included for the initial database. As described previously, this is likely to represent approximately 25% of all TBI admissions and 50% of all TBI admissions with ICP monitors [5]. Inclusion criteria were as follows: TBI; computerized invasive monitoring of ICP and ABP for at least 12 h; admission Glasgow Coma Scale (GCS); and 6-month mortality data available. From this database of 1146 patients, files were selected that contained an initial ICP less than 25 mm Hg with a subsequent ICP rise to over 40 mm Hg for at least an hour. This yielded 33 suitable files. Patients were managed according to TBI guidelines [6] aimed at keeping ICP < 20 mm Hg and CPP > 50–60 mm Hg. While all 33 patients in the cohort had computerized ICP monitoring, fewer patients had PRx monitoring (*n* = 24) or brain oxygenation monitoring (*n* = 9). This is because PRx monitoring only began in 1996 and brain oxygenation monitoring in 2004.

### Data Acquisition and Processing

ICP was monitored with an intraparenchymal sensor (Codman ICP MicroSensor, Codman & Shurtleff, Raynham, Massachusetts). ABP (Baxter Healthcare, Deerfield, Illinois) at the level of the right atrium (1992–2015) and at the foramen of Monro (2015–2017—one patient). Brain tissue oxygenation was monitored using a Licox probe via a cranial access device (Technicam, Abbott, UK). Probes were positioned at a constant depth in the white matter, pericontusional in focal injuries or in the non-dominant frontal lobe in diffuse injuries. Probe positioning was verified by means of a head computed tomography (CT) scan.

All data were sampled at least 50 Hz with proprietary data acquisition and analysis software (ICM 1992–2002 [7] and then with ICM+©, http://www.neurosurg.cam.ac.uk/icmplus after 2002). Heart rate was determined as the fundamental frequency of the ABP signal over a 10 s window within the cardiac (40–180 cycles/min) frequency band. Amplitude of the cardiac pulse in ICP and ABP was determined as the fundamental amplitude in the cardiac frequency band (40–180 cycles/min). ABP and ICP signals were averaged (mean) over a 10-s window; then PRx was calculated as the moving Pearson correlation of 30 consecutive ABP and ICP, updated every minute. Cerebrospinal compensatory reserve (RAP) was calculated similarly as the moving correlation between mean ICP and the pulse amplitude of ICP. AMP was divided by aABP (giving AMP/aABP ratio) to get an indication of the transmission of the cardiac pulse from the blood pressure to the ICP.

ICP, ICP pulse amplitude, ABP, ABP pulse amplitude, and RAP data were available for all 33 patients, while PRx and P_BT_O_2_ were available for 24 and 9 patients, respectively.

### Statistical Analysis

Data are reported as means and standard deviations. The relationship between minute-by-minute values of mean ICP and ICP pulse amplitude was fitted with a generalized additive model allowing for three different segments (cubic regression spline smooth). The number of segments (3) was chosen a priori to allow for 3 different portions of the ICP, AMP relationship (~ flat at low ICP, steep rising segment, and an upper breakpoint).

For the aggregate relationship between ICP or CPP and intracranial parameters (PRx, RAP, AMP), the means of each variable from each patient were calculated in 10 mm Hg wide intervals of ICP and CPP and then local regression smoothing (LOWESS) was applied. This binning procedure, prior to LOWESS fitting was to ensure that patients with longer recordings did not contribute disproportionately to parts of the smooth. Pairwise comparisons between physiological variables at the different ICP levels were performed using Student’s *t* test. No adjustments for multiple comparisons were made in this exploratory analysis. To test whether early PRx was different between those who developed refractory high ICP and TBI patients who did not, we first identified controls for the 24 cases who had simultaneous ICP and PRx data with 24 controls (selected from the 1146 patients) who were matched for sex, initial GCS and age. Then we performed a Wilcox test for the ICP values between the two groups. Because the primary aim of this study was descriptive, we used all available cases in the database rather than performing a priori sample size calculations. We used the R language and software environment for statistical computation (R Core Team 2015 version 2.12.1 [8]) using the following packages: dplyr [9], ggplot2 [10], gam [11], and MatchIt [12]. The significance level was set at 0.05.

## Results

Admission characteristics are displayed in Table 1. Of the 33 patients, 7 were female and the mean age was 30.3. In the cohort, ICP rose from a mean minimum of 5.46 (± 6.1) to a mean maximum of 74.67(± 22.76). Seventeen of the patients died.

**Table 1:** Patient demographics; cerebral effects of refractory intracranial hypertension after TBI

	Overall
*N*	33
Age (years) (mean (sd))	30.30 (12.54)
Sex = male (number, %)	26 (78.8)
GCS <= 8 (number, %)	28 (84.8)
Monitoring length (h) (mean (sd))	188.53 (145.40)
Decompressive craniectomy (number, %)	
No	15 (45.5)
Yes	11 (33.3)
NA	7 (21.2)
ICP (mm Hg) (mean (sd))	25.60 (8.71)
Max ICP (mm Hg) (mean (sd))	74.67 (22.76)
Min ICP (mm Hg) (mean (sd))	5.46 (6.10)
CPP (mm Hg) (mean (sd))	67.70 (10.98)
PRx (a.u.) (mean (sd); *n* = 24)	0.16 (0.25)
Mortality = dead (%)	17 (51.5)

*CPP* cerebral perfusion pressure, *GCS* Glasgow coma scale, *ICP* intracranial pressure, *PRx* pressure reactivity index

**Fig. 1:**
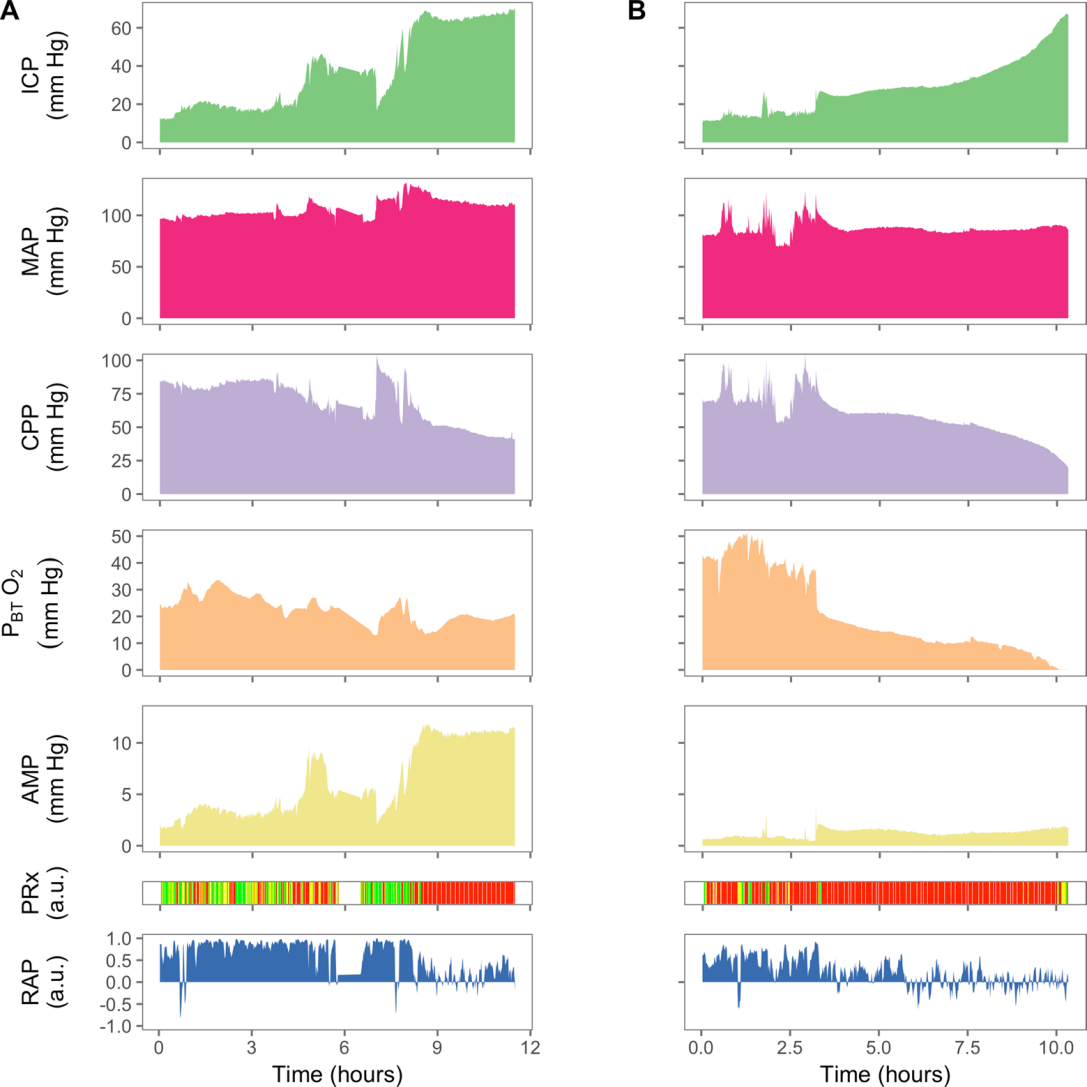
Neuromonitoring during severe intracranial hypertension in two traumatic brain-injured patients. On the left, ICP rises to 70 mm Hg over a period of 12 h and PRx was not significantly impaired prior to the increase in mean ICP. In addition, although P_BT_O_2_ decreased with increasing ICP, it did not reach severely hypoxic values even during the maximal mean ICP (P_BT_O_2_ ~ 20 mm Hg at ICP 60 mm Hg). On the right, ICP rises dramatically from below 20 mm Hg to over 60 mm Hg in the space of 10 h. This increase in ICP was associated with a fall in CPP, and brain tissue oxygenation. In this case, PRx was disturbed (> 0.25) even in the first 3 h, while ICP was under 20 mm Hg. Despite large increases in ICP over the last 5 h, pulse amplitude of ICP (yellow) shows little change. These two cases illustrate that refractory intracranial hypertension may have different neuromonitoring phenotypes. ICP intracranial pressure; AMP pulse amplitude of ICP; MAP mean arterial pressure; CPP cerebral perfusion pressure; P_BT_O_2_ brain tissue oxygenation; PRx pressure reactivity index; RAP index of cerebrospinal compensatory reserve

Two patient examples illustrating the heterogeneous nature of the response to intracranial hypertension are depicted in Fig. 1. In the patient in Fig. 1a, PRx is initially preserved and only becomes impaired after the development of raised ICP. In the other patient (Fig. 1b), PRx is clearly disturbed prior to the development of severe refractory raised ICP. In this case, a decrease in P_BT_O_2_ followed the decrease in CPP almost linearly to reach oxygen pressures of less than 5 mm Hg. In addition, a dissociation between the rise in mean ICP and pulse amplitude of ICP can be seen near the end of the recording, such that with increasing ICP, pulse amplitude remained constant. This failure for ICP pulse amplitude to increase despite increases in mean ICP has been postulated to be due to critical cerebrovascular collapse.

**Fig. 2:**
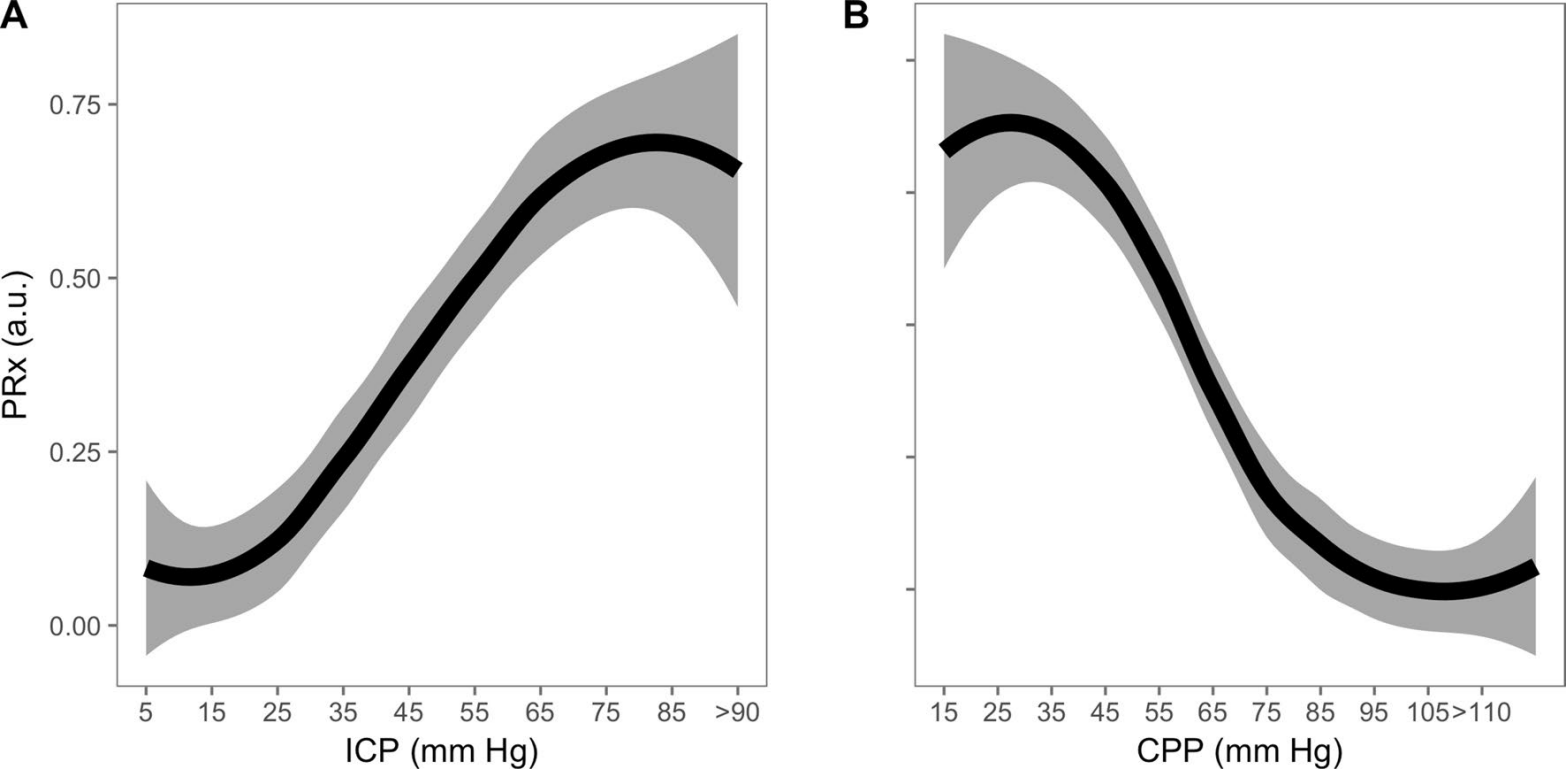
PRx response to refractory intracranial hypertension expressed relative to changes in ICP (left) and CPP (right) (LOWESS with 95% confidence interval; *n* = 24). Pressure reactivity increased with increasing ICP and PRx plotted against CPP revealed a partial ‘U-shaped’ curve as previously described. PRx is well maintained until CPP drops below 70 mm Hg, below which PRx deteriorates. PRx pressure reactivity; ICP intracranial pressure; CPP cerebral perfusion pressure

**Fig. 3:**
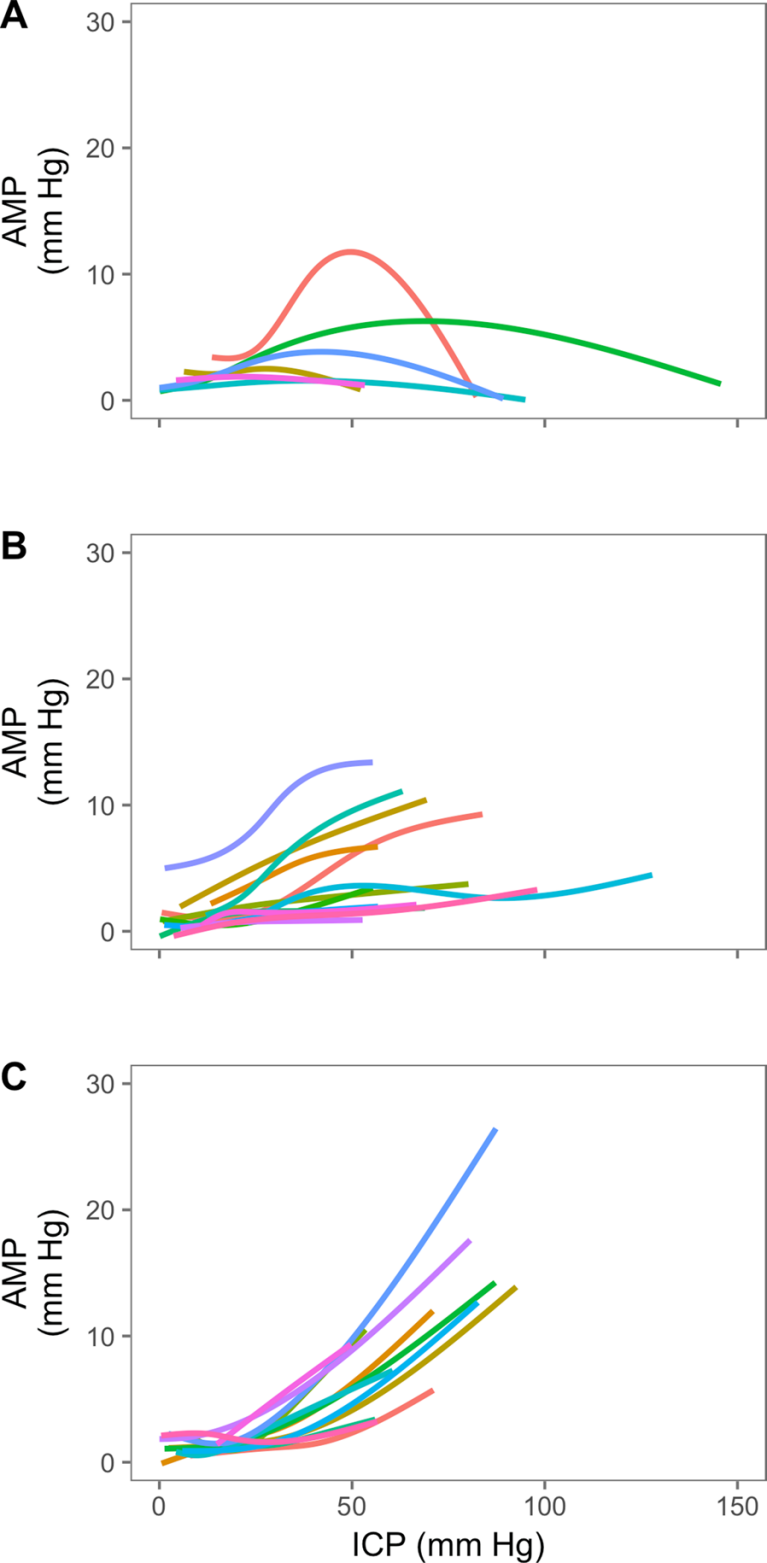
The ICP amplitude—mean ICP relationship (*n* = 33). Three distinct patterns were identified; those with an upper breakpoint (A, *n* = 6), a rightward deflected pattern (B, *n* = 13) or those with a monotonic increasing pattern (C, *n* = 12). In 2 patients, AMP decreased with increasing ICP (not shown). ICP intracranial pressure; AMP pulse amplitude of ICP

**Fig. 4:**
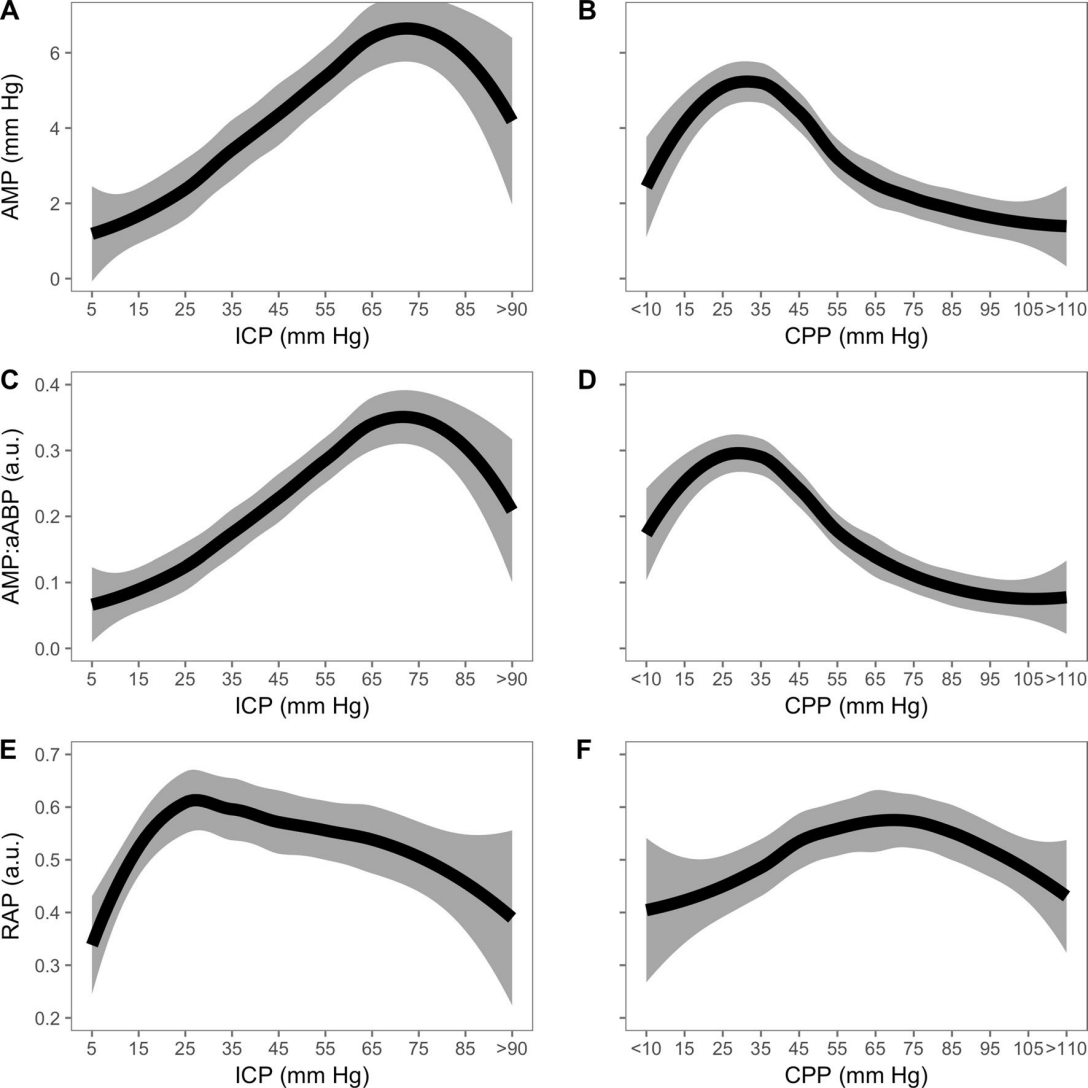
Relationship between ICP (left) or CPP (right) with ICP amplitude, transmission of arterial to intracranial pulse, and RAP (LOWESS with 95% confidence interval; *n* = 33). When all patients are grouped together, an upper breakpoint in the AMP–ICP relationship occurs at around 70 mm Hg. A similar response is seen for arterial to intracranial pulse transmission indicating that a decreased ABP amplitude is not responsible for the AMP-ICP upper breakpoint. RAP increases from low (0 mm Hg) to moderate ICP (~ 30 mm Hg) and thereafter decreases with further increase in ICP. ICP intracranial pressure; CPP cerebral perfusion pressure; RAP cerebrospinal compensatory reserve

Across all patients, PRx (Fig. 2) showed a general increasing trend, signifying disturbed autoregulation with increasing ICP and of note, the PRx at baseline levels of ICP is disturbed in a number of the patients (‘Appendix’ Fig. 7). As expected, PRx plotted against CPP reveals a steadily increasing PRx with decreasing CPP. The relationship between mean ICP (and CPP) levels and ICP pulse amplitude is depicted in Figs. 3 and 4. In general, ICP amplitude increased monotonically with increasing mean ICP (12 patients; Fig. 3c); however, in 6 of the cases an upper breakpoint (a switch from a positive to a negative relationship between mean ICP and ICP pulse amplitude) is seen at high ICPs (Fig. 3a). In 13 patients, a rightward deflection of the AMP–ICP was detected (Fig. 3b). In the remaining 2 patients, AMP monotonically decreased with increasing ICP (up until ICP 40 mm Hg—not included in figure). Similar responses were seen when ICP pulse amplitude was normalized to the arterial pulse amplitude (Fig. 4, middle panel). The index of cerebral compensatory reserve, RAP, increased from low to moderate levels of ICP and thereafter showed a gradual decline with further increases in ICP (Fig. 4e). At all levels of ICP or CPP, the averaged RAP response was greater than + 0.3 a.u.

**Fig. 5:**
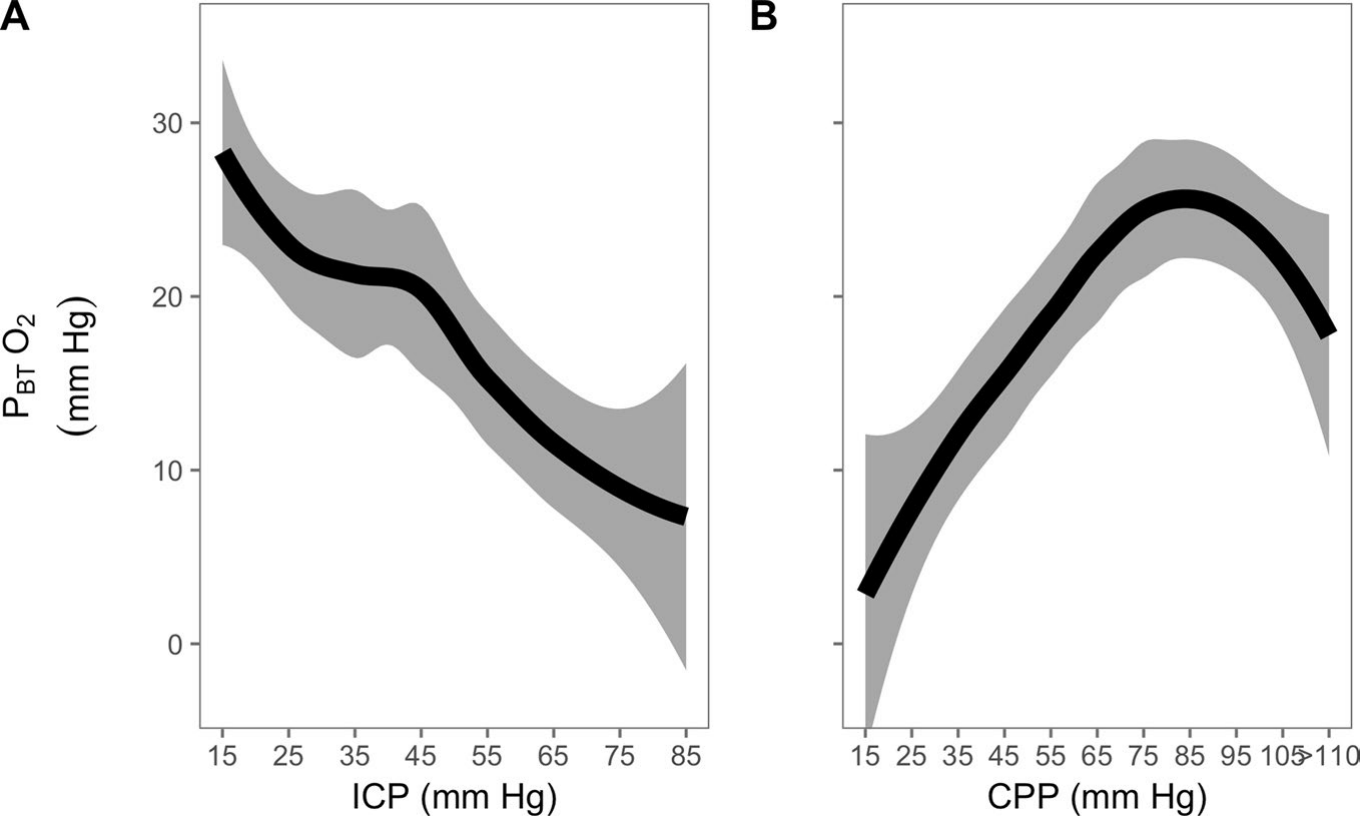
P_BT_O_2_ response to refractory intracranial hypertension expressed relative to changes in ICP (left) and CPP (right) (LOWESS with 95% confidence interval; *n* = 9). When expressed against ICP, P_BT_O_2_ demonstrates a steady decrease. When expressed in relation to changes in CPP, the relationship resembles the autoregulation curve; with moderate levels of CPP (70- > 90 mm Hg), oxygenation is well maintained, but lower than 70 mm Hg, oxygenation decreases (by approximately 0.5 mm Hg per 1 mm Hg decrease in CPP. P_BT_O_2_ brain tissue oxygenation; ICP intracranial pressure; CPP cerebral perfusion pressure

When all 9 available P_BT_O_2_ responses are viewed together (Fig. 5), P_BT_O_2_ shows a steady decrease with increasing ICP. However, the between patients response is strikingly variable (Fig. 8 ‘Appendix’). When P_BT_O_2_ is plotted against CPP, a consistent pattern is seen, with a relatively preserved P_BT_O_2_ with mild decreases in CPP followed by a steeper decrease with moderate to severe decreases in CPP. All but one patient shows a decrease in P_BT_O_2_ with decreasing CPP.

**Fig. 6:**
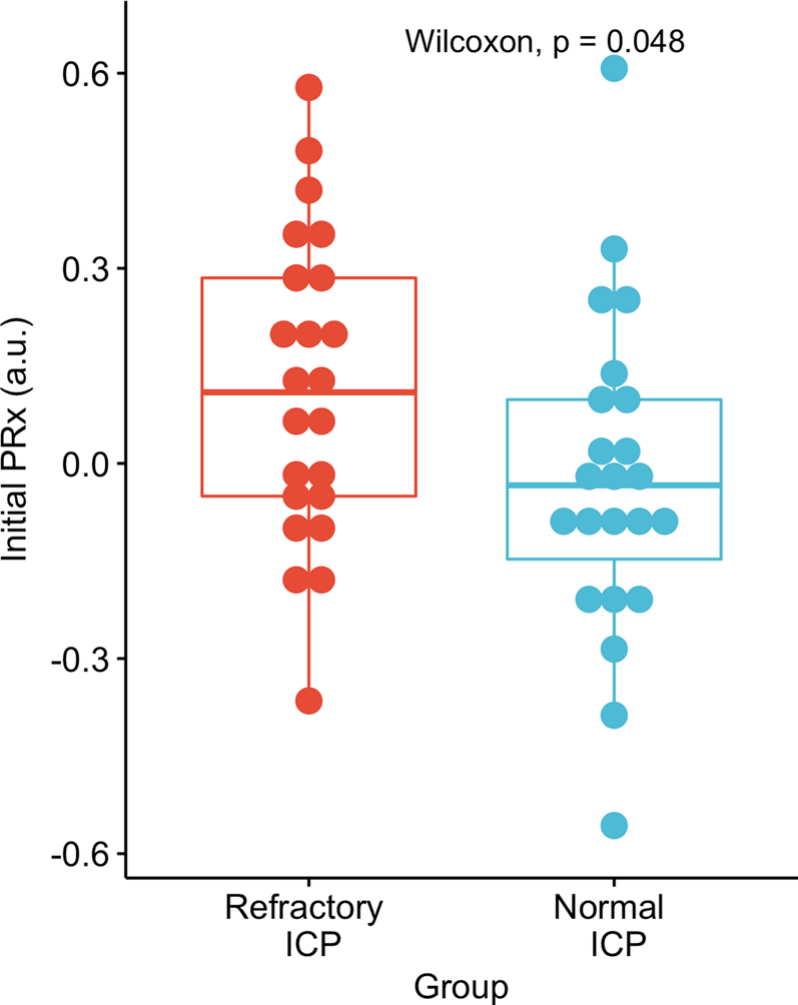
PRx over the first 5 h in patients who went on to develop severe refractory intracranial hypertension (*n* = 24) compared to severe TBI patients matched for age, sex and initial GCS (*n* = 24). Those who developed severe refractory intracranial hypertension tended to have higher PRx in the first 5 h of monitoring (*p* = 0.048) while ICP was not significantly different between the two groups (*p* = 0.197). PRx pressure reactivity index; ICP intracranial pressure

**Fig. 7:**
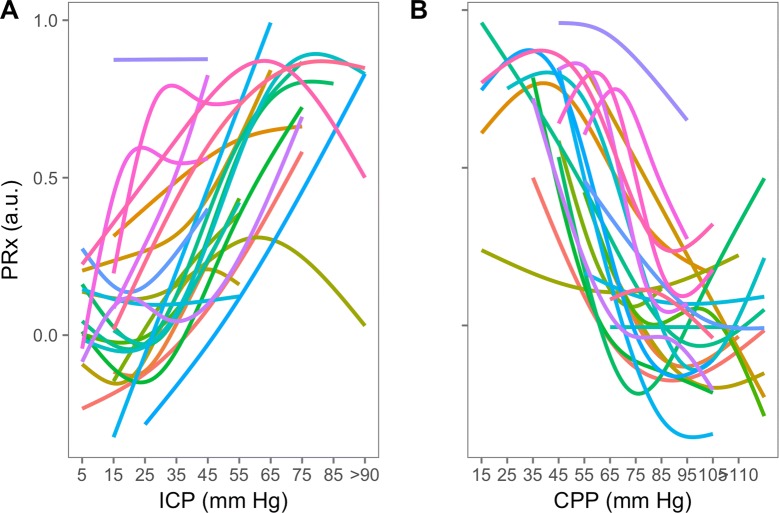
Individual PRx responses to refractory intracranial hypertension expressed relative to changes in ICP (left) and CPP (right) (*n* = 24). While pressure reactivity index increased with increasing ICP in each patient, in some patients, PRx was disturbed (above > 0.25) even at relatively normal ICP (below 25 mm Hg). PRx plotted against CPP revealed a partial ‘U-shaped’ curve in most patients, as previously described. PRx is well maintained until CPP drops below 70 mm Hg, below which PRx deteriorates. PRx pressure reactivity; ICP intracranial pressure; CPP cerebral perfusion pressure

**Fig. 8:**
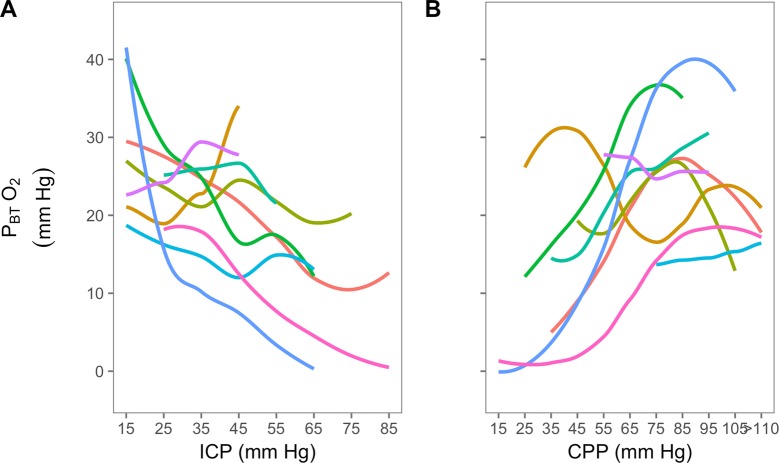
Individual P_BT_O_2_ response to refractory intracranial hypertension expressed relative to changes in ICP (left) and CPP (right) (*n* = 9). Brain tissue oxygenation response to increases in ICP showed marked variability between patients with some patients showing relatively preserved P_BT_O_2_ with increasing ICP, some showing precipitous hypoxia, and some showing a paradoxical increase in oxygenation. A similar variability is observed with respect to CPP. This variability highlights the complex nature of the P_BT_O_2_ signal; it is contributed to by respiratory (FiO_2_, PaO_2_), hematological (Hb), cardiovascular (cardiac output, cerebral blood flow) and metabolic factors (brain metabolism)

When compared to 24 controls (severe TBI patients, matched for age, sex, and initial GCS), initial mean PRx in the first five hours of monitoring was higher (Fig. 6) in the cases of refractory intracranial hypertension. While ICP was also higher, this did not reach statistical significance (Wilcox *p* = 0.197).

Grouping each of the patients’ data into three ICP groups (< 25, 25–50, > 50 mm Hg; Table 2) confirmed the visual interpretation of Figs. 2, 3, and 4).

**Table 2:** Physiologic response to high intracranial pressure after TBI (mean (sd); *n* = 33 unless stated otherwise)

Variable	0–25 mm Hg	25–50 mm Hg	50–150 mm Hg	*p* (elevated vs. base)	*p* (severe vs. elevated)
ICP (mm Hg)	17.52 (3.85)	32.80 (2.39)	61.04 (10.79)	< 0.001	< 0.001
AMP (mm Hg)	1.83 (1.36)	3.10 (2.08)	5.34 (4.26)	0.02	< 0.001
AMP/aABP (a.u.)	0.10 (0.06)	0.17 (0.10)	0.29 (0.22)	0.02	< 0.001
CPP (mm Hg)	73.90 (9.27)	63.79 (10.41)	39.27 (18.11)	< 0.001	< 0.001
MAP (mm Hg)	91.42 (9.87)	96.61 (9.48)	99.82 (12.55)	0.01	0.120
aABP (mm Hg)	18.87 (3.32)	19.70 (4.21)	19.85 (5.85)	0.16	0.712
HR (bpm)	77.53 (15.35)	79.46 (19.04)	83.78 (20.72)	0.50	0.214
PRx (a.u.) (*n* = 24)	0.06 (0.26)	0.21 (0.30)	0.57 (0.24)	0.01	< 0.001
RAP (a.u.)	0.54 (0.20)	0.59 (0.25)	0.46 (0.27)	0.29	0.005
P_BT_O_2_ (mm Hg) (*n* = 9)	27.27 (7.32)	20.78 (5.43)	12.68 (7.09)	0.06	0.02

*ICP* intracranial pressure, *AMP* pulse amplitude of ICP, *aABP* amplitude of arterial blood pressure, *MAP* mean arterial pressure, *CPP* cerebral perfusion pressure, *HR* heart rate, *PRx* pressure reactivity index, *RAP* cerebrospinal compensatory reserve, *P*_*BT*_*O*_*2*_ brain tissue oxygenation

## Discussion

By isolating the rare cases of severe refractory intracranial hypertension with multimodality monitoring, we describe the cerebral physiological response to raised ICP after TBI. Although only exploratory, these data highlight the impact of raised ICP on cerebral autoregulation, the sensitivity of brain tissue oxygenation to raised ICP and the possible role of impaired pressure reactivity in identifying at-risk patients.

### Autoregulation Parameters

Raised ICP impairs dynamic cerebral autoregulation (Fig. 2, Table 2). This confirms previous investigations of autoregulation during short-term increases in ICP (plateau waves) [13, 14] and is consistent with a recent large between patient analysis that found a significant correlation between mean ICP over the whole monitoring period and mean PRx [15]. In contrast to these studies, however, the current analysis observed changes *within* patients and over periods of time that are substantially longer than the calculation window for PRx. Therefore, the finding of impaired PRx with increasing ICP is unlikely to be explained by between patient confounding factors or time-resolution limitations of the PRx method. Similarly, PRx deteriorates with decreased CPP, representing the lower half of the ‘U-shaped’ relationship between CPP and PRx [16].

Perhaps most striking though, is that PRx was disturbed before the onset of intracranial hypertension in a selection of the patients. This has been observed previously in a cohort of brain-injured patients of mixed pathologies (subarachnoid hemorrhage, hypoxic brain injury, trauma), but has not been statistically assessed [17]. After matching cases of severe refractory high ICP for age, initial GCS, and sex, we found that PRx in the first 5 h was higher in those who developed raised ICP. The same was not true for the mean ICP over the first 5 h, although this did approach statistical significance (*p* = 0.197). This highlights the potential utility of continuous assessments of cerebral autoregulation in predicting raised ICP events. Encouragingly, a previous investigation found that a feature similar to PRx (the long term mean arterial pressure (MAP)-ICP correlation from low-frequency data) can help predict periods of raised ICP 30 min before they occur [18]. Identifying patients at risk of raised ICP may prove crucial as raised ICP carries a high risk of mortality, and perhaps the most effective ICP treatments, such as decompression, require some time to mobilize.

Reasons why impaired cerebral pressure reactivity may be a harbinger of raised ICP in some patients but not others deserves further discussion as it perhaps reflects different pathophysiological profiles of intracranial hypertension. Intracranial hypertension is a result of changes in compartmental volumes within the brain: increases in volume of the brain tissue (for example cerebral edema), the cerebral spinal fluid, or the blood (venous or arterial). Disturbed autoregulation preceding an increase in ICP may indicate a primary vascular dysfunction that, over time, causes raised ICP. In these cases, a vascular dysfunction as manifest in impaired PRx could lead to vasogenic edema, which over hours could lead to progressive increases in ICP. Cases where ICP and PRx are disturbed simultaneously indicate that either the impaired vascular function is a consequence of the raised ICP, or the underlying pathophysiological mechanism is occurring rapidly. Simultaneous perturbations in PRx and ICP could conceivably occur with obstruction in CSF flow, a rapidly expanding lesion or during a sudden increase in blood volume.

In addition, we found a consistent increase in MAP at elevated levels of ICP (Table 1. While previous experimental work has shown profound Cushing vasopressor responses to extreme and rapid increases in ICP [2, 19], in these data we cannot rule out a confounding effect of concurrent interventions and medications such as cooling or vasopressors.

### Brain Oxygenation

In this sample of patients with extreme increases in ICP, P_BT_O_2_ overall decreased. This is consistent with a recent experimental study which demonstrated a cortical vulnerability to increased ICP [4]. However, there was marked variation between patients in the levels of P_BT_O_2_ and the response to increased ICP. This highlights the complex nature of the P_BT_O_2_ variable. It does not merely index brain perfusion, but the complicated interplay between cerebral oxygen delivery (dependent on P_a_O_2_, Hb, CBF), metabolic rate of the nearby cerebral tissues, and any diffusion barriers [20–22]. Therefore, many clinical scenarios, potentially independent of perfusion, may affect brain oxygenation such as red blood cell transfusion, hypoxia or hyperoxia, mitochondrial dysfunction [23–25]. The position of the oxygen sensing probe may also be relevant as intracontusional oxygen monitoring should be different to pericontusional or healthy tissue monitoring [26]. Furthermore, the depth of probe may also be relevant; in basilar artery dependent rabbits with CSF infusion induced increases in ICP, the cortical laser Doppler flux was more sensitive to increases in ICP than the global basilar artery flow velocity [4].

Nevertheless, monitoring of brain oxygenation, sometimes in combination with cerebral microdialysis has increased in popularity over last decade with some promising initial results. In a retrospective analysis comparing before P_BT_O_2_ to after P_BT_O_2_-targeted therapy, found that keeping P_BT_O_2_ above 20 mm Hg was associated with decreased mortality [27]. In addition, similar to PRx, brain tissue monitoring has been combined with ABP and CPP monitoring to yield continuous autoregulation assessment in TBI and subarachnoid hemorrhage [28–30].

### The Mean ICP and ICP Pulsatile Amplitude Relationship

The increase in pulsatility of ICP with increasing levels of mean ICP has been interpreted as a loss of cerebral compensatory reserve. In this scenario, when the intracranial system is on the steep ascending portion of the pressure–volume curve, a pulsatile injection of blood volume from the cardiac cycle would be expected to produce a large increase in pulsatile pressure. However, at extreme levels of ICP (approaching diastolic blood pressure) it has been demonstrated in animal models that ICP pulsations may in fact decrease [31]. This has been proposed to be related to critical closing of the cerebrovascular bed [7]. Observing this phenomenon is, however, far from universal in refractory intracranial hypertension (Fig. 3), probably as its occurrence is multifactorial, depending on parameters such as vasomotor tone, cerebral intravascular pressures and arterial blood pulse pressure. Interestingly, RAP—the short-term correlation between changes in mean ICP and mean amplitude of ICP—did not show a similar upper breakpoint like the pulsatility–mean ICP relationship and rarely reached negative values (Fig. 4 and 1) as may have been expected at these extreme levels of ICP [32]. This perhaps reflects the short-term dynamic nature of the RAP calculation (calculated over a 5-min time window) compared with the extended time windows associated with the pulsatility-mean ICP relationship.

### Limitations

Due to the small sample size, these analyses must be considered as preliminary descriptions. While it was possible to include more patients by relaxing the definition of refractory intracranial hypertension, we wished to describe the physiological response across the widest range of ICPs and therefore opted to only include patients with severe intracranial hypertension. Further, because intracranial physiology will depend heavily on concurrent therapies, detailed clinical annotations to the monitoring data would aid interpretation and increase the generalizability of the study. Stratification of physiological responses to intracranial hypertension by ICP treatment modality (for example decompression or barbiturates) may yield useful information describing early indicators of a beneficial versus a pathological physiological response. The association between early PRx and later development of intracranial hypertension needs to be treated with caution as the case–control analysis was only carried out in a small subset of patients with concurrent PRx monitoring (*n* = 24). Furthermore, time points besides the first 5 h for the ability of PRx to predict intracranial hypertension should be investigated. In addition, the current study does not address the pragmatic issue of how multimodality monitoring data can be integrated into clinical care without causing a detrimental information overload. While ongoing data collection with the increasing availability of multimodality monitoring will likely shed light on this area in the near future, controlled experimental studies that mimic the increase in ICP observed after human TBI need to also play a role as they can effectively isolate the effects of the disease from that of treatment.

## Conclusion

Severe intracranial hypertension after TBI leads to decreased brain oxygenation, impaired pressure reactivity, and a characteristic ICP pulsatility response.

## Appendix

See Figs. 7 and 8.
